# Syphilis may be a confounding factor, not a causative agent, in syphilitic ALS

**DOI:** 10.12688/f1000research.9318.1

**Published:** 2016-08-02

**Authors:** Bert Tuk

**Affiliations:** 1 Leiden Academic Center for Drug Research (LACDR), Leiden University, Leiden, 2333 CC, Netherlands; 2 Ry Pharma, Hofstraat 1, Willemstad, 4797 AC, Netherlands

**Keywords:** amyotrophic lateral sclerosis, ALS, syphilitic ALS, GABA antagonist, penicillin G, benzylpenicillin, hydrocortisone, circadian rhythm

## Abstract

Based upon a review of published clinical observations regarding syphilitic amyotrophic lateral sclerosis (ALS), I hypothesize that syphilis is actually a confounding factor, not a causative factor, in syphilitic ALS. Moreover, I propose that the successful treatment of ALS symptoms in patients with syphilitic ALS using penicillin G and hydrocortisone is an indirect consequence of the treatment regimen and is not due to the treatment of syphilis. Specifically, I propose that the observed effect is due to the various pharmacological activities of penicillin G ( *e.g*., a GABA receptor antagonist) and/or the multifaceted pharmacological activity of hydrocortisone. The notion that syphilis may be a confounding factor in syphilitic ALS is highly relevant, as it suggests that treating ALS patients with penicillin G and hydrocortisone—regardless of whether they present with syphilitic ALS or non-syphilitic ALS—may be effective at treating this rapidly progressive, highly devastating disease.

## Introduction

Amyotrophic lateral sclerosis (ALS, also known as Lou Gehrig’s disease) is a devastating disease with an average post-diagnosis life expectancy of 3–5 years. The clinical manifestations of ALS are well described and include the progressive wasting of muscle mass, reduced muscle coordination, dysphagia, dysarthria, and fatal respiratory depression ^1– 3^. Several observations regarding the putative pathogenesis of ALS have been reported ^1– 3^; however, although more than 130 years have passed since it was first described, the pathogenesis of ALS remains poorly understood.

One of the many unresolved mysteries surrounding the pathogenesis of ALS is the presumed association of ALS with syphilis (so-called “syphilitic ALS”). The most puzzling observation in syphilitic ALS is that it is the only form of ALS that has been reported to have been treated effectively; specifically, in 1990 El Alaoui-Faris reported five cases of syphilitic ALS in which the patients were followed for 5–13 years after receiving treatment for syphilis ^4^. Interestingly, these five reports originated from one neurology center in Morocco. In this center, five patients tested positive for syphilis in their serum and/or cerebrospinal fluid (CSF) and were successfully treated using a specific combination of penicillin G and hydrocortisone, the standard of care for neurosyphilis in Morocco at the time. Because their ALS symptoms also resolved during treatment, and because these patients no longer tested positive for syphilis, the group concluded that the patients’ ALS symptoms were caused by syphilis, and they concluded that neurosyphilis can lead to ALS, giving rise to the syphilitic ALS hypothesis ^4^.

Here, I postulate that in countries in which syphilis is highly endemic, syphilis may actually be a confounding factor in syphilitic ALS, and this factor should be considered carefully before drawing conclusions regarding the efficacy of treatment in these populations. Based upon a careful review of the aforementioned five reported cases of syphilitic ALS, I conclude that the only evidence for the existence of syphilitic ALS as a specific disease entity stems from fact that the patients’ ALS symptoms resolved simultaneously with the shift from syphilis-positive to syphilis-negative following treatment with penicillin G and hydrocortisone. Thus, I propose the novel hypothesis that the successful treatment of syphilitic ALS with a specific penicillin G and hydrocortisone regimen is independent of the treatment of syphilis. Specifically, I propose that the anti-syphilis treatment treated these patients’ ALS directly via the off-target pharmacological activity of penicillin G ( *e.g*., as a GABA receptor antagonist) and/or hydrocortisone.

## Case reports of five patients with syphilitic ALS who were followed for 5–13 years post-treatment

The five reported cases of treated syphilitic ALS published in 1990 are summarized in Table 1 and Table 2 ^4^. These cases originated from a neurology center in Morocco that was highly experienced in the diagnosis of ALS ^4^. Out of a total of 40 ALS patients who were diagnosed at this neurology center, five were diagnosed with syphilitic ALS. The age at onset of ALS was 27–48 years, and the clinical symptoms included classic features of ALS, including functional impairment of the upper limbs in three patients and spastic paraparesis in two patients ^4^. Before receiving treatment, progressive neurological dysfunction had been present from several months to up to 3 years. Two patients also displayed symptoms that are not typically present in ALS patients: patient 3 reported sudden regressive deafness, which was later found to be caused by meningogenic labyrinthitis, and patient 5 had horizontal nystagmus.

**Table 1.** Demographic summary reported cases on syphilitic ALS ^4^.

Case	Sex	Age at syphilis onset (years)	Age at ALS diagnosis (years)	Treatment outcome	Follow-up (years)
1	F	16	39	Stabilized	13
2	M	No history of syphilis	37	Full recovery	8
3	M	unknown	40	Good recovery	8
4	M	No history of syphilis	27	Good recovery	5
5	M	No history of syphilis	48	Mild recovery	7

**Table 2.** Summary of ALS symptoms in five reported cases of syphilitic ALS ^4^.

	Case 1	Case 2	Case 3	Case 4	Case 5
Upper limb atrophy	+	+	+	+	+
Muscle fasciculation	+	+	+	+	+
Tongue fasciculation	+	–	–	–	–
Muscle cramps	–	+	+	–	+
Tendon reflexes	+	+	+	+	+
Bulbar symptoms	+	–	–	–	–
EMG denervation	+	+	+	+	+
Normal motor conduction velocity	+	+	+	+	+
Babinski response present	+	–	+	+	–

**Table 3.** 21-day treatment regimen reported to provide long-term reversal of symptoms in syphilitic ALS, when administered every 3 months for four times ^4^.

Day(s)	8 hour continuous infusion
1	1 million units penicillin G + 100 mg hydrocortisone
2	3 million units penicillin G + 100 mg hydrocortisone
3	5 million units penicillin G + 100 mg hydrocortisone
4	10 million units penicillin G + 100 mg hydrocortisone
5	20 million units penicillin G + 100 mg hydrocortisone
6–14	20 million units penicillin G + 100 mg hydrocortisone
15–21	20 million units penicillin G

## Discussion and conclusions regarding syphilitic ALS

Syphilitic ALS is an intriguing phenomenon, as it is the only form of ALS ever reported to have been cured ^4^. Moreover, syphilitic ALS has only been reported in Morocco, a country in which syphilis is highly endemic, thereby greatly increasing the likelihood of presenting in patients with ALS. Lastly, neurosyphilis can be a major confounding factor when diagnosing ALS. Therefore, these five case reports of putative syphilitic ALS were reanalyzed in order to explore plausible explanations for these patients’ resolution of ALS symptoms.

First, consider the evidence supporting the existence of syphilitic ALS, which stems solely from the observation that ALS symptoms resolved after treatment with penicillin G and hydrocortisone in patients who also tested positive for syphilis. Because a cure for ALS had never been reported, and because penicillin G is an effective treatment for syphilis and neurosyphilis, the authors concluded that these patients had syphilitic ALS. Their reasoning was based on the fact that the treatment regimen—in addition to resolving the patients’ ALS symptoms—also treated the patients’ latent syphilis infections, as all five patients were negative for syphilis following treatment. Furthermore, the observation that syphilitic ALS has only been reported in Morocco may be explained by the striking fact that at the time, approximately 10% of the population in Morocco was positive for syphilis ^5^. Therefore, these observations appeared to support the notion that syphilis can cause ALS, giving rise to the syphilitic ALS hypothesis.

Here, however, I cast doubt on the notion that syphilitic ALS is a bona fide form of ALS and suggest that syphilis may in fact be a confounding factor. First, if syphilis can cause ALS, one would expect the prevalence of ALS to be higher in countries in which syphilis is highly prevalent. However, this has not been observed. During the period in which the five cases of syphilitic ALS were reported, the prevalence of syphilis was 300-fold higher in Morocco than in developed countries ^5^, yet the prevalence of ALS was similar between Morocco and the developed countries.

A second observation is that the ALS symptoms in these five patients either stabilized or resolved after the patients received increasing doses of penicillin G and hydrocortisone. From this perspective, it is important to note that both penicillin G and hydrocortisone have pharmacological profiles that extend beyond their pharmacotherapeutic applications for treating syphilis. For example, in addition to its antibacterial properties, penicillin G is also a potent antagonist of GABAergic ( *i.e*., inhibitory) activity ^6^ and can induce seizures in patients with renal insufficiency ^7^. Interestingly, GABAergic overstimulation was postulated recently to play a role in the pathogenesis of ALS ^8^. Furthermore, at sufficient dosages, penicillin G can affect several major bodily functions and/or systems, including the immune system, the cardiovascular system, metabolic function, renal function, liver function, the hematological system, and the urogenital system ^7^.

Similarly, hydrocortisone is a known immunomodulatory and anti-inflammatory agent ^9^, thus potentially affecting systems that are involved in the pathogenesis of ALS ^1– 3^. Moreover, hydrocortisone has reported efficacy in multiple sclerosis and respiratory diseases ^9^, conditions that have clinical overlap with ALS. Furthermore, like penicillin, hydrocortisone can affect several bodily functions and/or systems, including the endocrine system, the immune system, inflammatory function, the respiratory system, the hematological system, and the gastrointestinal system ^9^. Furthermore, hydrocortisone has been reported to affect the GABAergic system ^10^, the GABAergic system has been reported to affect the release of cortisone, the natural form of hydrocortisone ^11^. and glucocorticoids have been shown to be efficacious in preclinical models for ALS ^12^.

A third key observation is that syphilis represents a major confounding factor with respect to the interpretation of clinical outcome, particularly in countries in which syphilis is highly prevalent. The neurology center where the five cases of syphilitic ALS were identified in the 1980s reported that these five patients were identified from a total of 40 patients with ALS. This corresponds to a 12.5% prevalence of syphilis among ALS patients in this neurology center. Strikingly, this percentage is on par with the prevalence of syphilis in the general population of Morocco ( *i.e.*, 10%) in that same time period ^5^. Given this extremely high prevalence, syphilis (particularly neurosyphilis) is likely a confounding factor in the interpretation of many clinical conditions, including neuromuscular diseases such as ALS.

Based on these observations and potential confounding factors, interpreting the clinical outcome in these five patients with syphilitic ALS can lead to incorrect conclusions, unless this treatment is also tested in ALS patients who are negative for syphilis. Given that this has not been attempted, the potential confounding role of syphilis in syphilitic ALS allows for the possibility of alternative explanations. Therefore, I hypothesize that syphilis is actually a confounding factor in syphilitic ALS, and the apparent treatment of syphilitic ALS is actually due to the coincident treatment of both syphilis and ALS due to two separate therapeutic actions of the treatment regimen. I therefore propose that penicillin G and/or hydrocortisone may be effective at treating both syphilitic ALS and non-syphilitic ALS. If correct, this may have long-reaching implications for the treatment of ALS, a currently incurable disease. 

Support for this hypothesis comes from a 2013 report of a patient with syphilitic ALS ^13^. Given the relatively brief follow-up of this patient (less than one year), this case was not included in the current analysis. However, following a treatment regimen similar to treatment applied in the previous five cases, this patient was reportedly cured of ALS.

Other explanations may also account for the observations discussed above. First, some of the five initial patients who were treated for syphilitic ALS may have been misdiagnosed. However, this is unlikely, given that the neurology center had extensive experience diagnosing both ALS and syphilis ^4^. Furthermore, the symptoms associated with ALS ^1– 3^ generally do not overlap with the symptoms associated with syphilis ^5^. Secondly, it is possible that the patients’ ALS symptoms were caused by an infection other than syphilis, and this other infection was treated by the penicillin and hydrocortisone. Moreover, the presence of a previously unidentified infection—and its treatment with penicillin G—may explain the observation that the 21-day treatment (see Table 1) provided long-term reversal of symptoms ( *i.e*., up to 13 years). However, this is unlikely, as it would mean that all five patients had the same infection in addition to testing positive for syphilis. Most importantly, penicillin (a GABA receptor antagonist) may provide long-term treatment of ALS through an action other than its antibacterial activity. Precedence for this hypothesis comes from reports that other GABA receptor antagonists provide long-term effects by resetting the master circadian clock ^14^. Moreover, hydrocortisone is also reported to affect circadian rhythm ^15^.

In summary, given the published clinical observations, I propose that syphilis may actually be a confounding factor—not a causative agent—in the clinical entity currently called syphilitic ALS. The notion that syphilis may be a confounding factor in syphilitic ALS is highly relevant, as it suggests that treating patients with ALS—regardless of whether they have syphilitic ALS or non-syphilitic ALS—may be effective at slowing or even reversing the progression of ALS. Based on the rapidly progressive and highly devastating nature of ALS, further research is needed to test this novel hypothesis, possibly leading to the first effective treatment for ALS and perhaps other progressive neuromuscular diseases.
