# Supplementary material for: Syphilis may be a confounding factor, not a causative agent, in syphilitic ALS
Source: F1000Res. 2016 Aug 2;5:1904. [Version 1] doi: 10.12688/f1000research.9318.1 (PMC5081158; doi:10.12688/f1000research.9318.1)
Supplement: Peer review history

# Peer review: Referee response for version 1

**DOI:** 10.5256/f1000research.10034.r16950

**Published:** 2016-10-21

**Authors:** Alan Gill

**Affiliations:** 1 ALS Therapy Development Institute, Cambridge, MA, USA

**Introduction**

 The antecedent hypothesis for this work might be summarized in this way: excitatory overstimulation is a homeostatic response to inhibitory overstimulation. What is actually needed is to reduce inhibitory activity (the primary problem) so compensatory excitatory overstimulation is no longer required.

 Given the widely accepted working model that reduced inhibitory and excessive excitatory neuronal activity contributes to ALS through neuronal excitotoxicity, the hypothesis asks that we look at ALS with new eyes. In this devastating disease for which we have no satisfactory treatment, looking with new eyes is certainly warranted.

 The current work provides evidence that co-treatment with PENG and hydrocortisone beneficially slows ALS progression, even in ALS patients that did not have syphilis. The current work’s hypothesis might be summarized in this way: PENG helps ALS symptoms because it is a GABA receptor antagonist. Hydrocortisone helps with inflammation also present in ALS.

 Apart from the limitations of our understanding ALS pathology, the current work opens an array of complexities, including regulatory interneuron networks, inhibitory receptor subtypes, antagonist drug properties and side-effects, neuronal ion channel composition, neuropeptide modulation, central pattern generator function, and neuroinflammation. For example, why tiptoe into GABA antagonism with PENG when more refined and specific inhibitors are available. Or, patient-to-patient variability in blood-brain barrier leakiness could make it difficult to control CNS PENG levels and the probability of seizure induction. Or, should somatostatin-expressing interneuron networks be selected for in our treatment approach? Should studies include cohorts that do not receive hydrocortisone, or only hydrocortisone? Combination drug studies, if anything is to be learned from them, require expensive designs that test each drug separately and the identification of a basis for doses used in the combination, or combinations.

 The complexity is formidable. But in the end, what is really important is whether PENG plus hydrocortisone actually slows ALS progression, i.e. “does it work?”. Even if we don’t understand why we have an obligation to try what works. The true purpose of understanding the complexities above is to try to design a reliable therapeutic regimen for clinical application. We want the therapy to work predictably and we don’t want it to do harm.

 The comments and references that I have compiled below are intended to stimulate the process of making the potential therapy safe and predictable.

**Comments and References:**

 The outcome of homeostatic regulation depends on the complement of ion channels expressed in cells: in some cases, loss of specific ion channels can be compensated; in others, the homeostatic mechanism itself causes pathological function ^1^.

 There may be a need to target restricted GABAergic subpopulations and cell types characterized by distinct laminar location, morphology, axonal projection, and electrophysiological properties ^2^.

**Increasing GABA May Be Helpful**

 The GABA analog Pregabalin increases extracellular GABA by inducing its synthesis and transport. Cacna2d2, the gene encoding the Alpha2delta2 subunit of voltage-gated calcium channels (VGCCs), is a developmental switch that limits axon growth and regeneration. In vivo, Alpha2delta2 pharmacological blockade through Pregabalin administration enhanced axon regeneration in adult mice after spinal cord injury. Since Pregabalin is already an established treatment for a wide range of neurological disorders, targeting Alpha2delta2 may be a novel treatment strategy to promote structural plasticity and regeneration following CNS trauma ^3^.

 Electrophysiological and histological studies support the pathophysiological concept of an impaired inhibitory, namely GABAergic, control of the motoneurons in the cerebral cortex of ALS patients. However, in the prefrontal and temporal cortex of ALS patients the GABA synthesizing enzyme glutamic acid decarboxylase (GAD) was significantly upregulated ^4^.

 There are reduced levels of GABA in the motor cortex of patients with ALS. Patients with ALS had significantly lower levels of GABA in the motor cortex than did healthy controls. Riluzole-naive patients with ALS had higher levels of glutamate-glutamine than did riluzole-treated patients with ALS or healthy controls ^5^^,^^6^.

   

**Increasing Glycinergic Function May Be Helpful**

 The selective loss of inhibitory glycinergic regulation of motoneuron function or glycinergic interneuron degeneration contributes to motoneuron degeneration in amyotrophic lateral sclerosis ^7^.

 Anti-glutamate drugs don’t have major disease-modifying effects clinically in ALS. Nevertheless, hyperexcitability of upper and lower motor neurons is a feature of human ALS and transgenic (tg) mouse models of ALS. Motor neuron excitability is modulated by presynaptic glycinergic and GABAergic innervation and postsynaptic glycine and GABA(A) receptors that mediate synaptic inhibition. Inhibitory glycinergic innervation of spinal motor neurons becomes deficient before motor neuron degeneration is evident in G93A-hSOD1 mice. GABAergic innervation of ALS mouse motor neurons and GABA(A) receptor function appear normal. Abnormal synaptic inhibition resulting from dysfunction of interneurons and motor neuron Glycine receptors may participate in ALS pathogenesis ^8^.

 Muscle paralysis during REM sleep requires activation of both GABA and glycine receptors together. Glycine alone doesn’t paralyze the muscles; GABA is also required. To prevent muscle paralysis during REM sleep both types of receptors have to be off. GABA antagonists like bicuculline, metrazol, and flumazenil would remove the GABA effect and reverse paralysis, stimulating muscle movement. In general these drugs produce stimulant and convulsant effects, and are mainly used for counteracting overdoses of sedative drugs. There is no clear way to direct their activity in a specific direction to solve ALS paralysis. GABA interneurons are part of most neuronal networks in the brain and spinal cord. They act to modulate activity through the networks and they do so by monitoring activity level and responding to the level in a way that keeps it within normal limits. They participate in rhythm and pattern generating networks that coordinate many kinds of brain and spinal cord activity, including breathing and movement.

 GABA loss seems to be less influential than glycine loss. In mice lacking physiological levels of GABA, there are distinct regional changes in motor neuron number and muscle innervation, which appear to be linked to their physiological function and to their rostral-caudal position within the developing spinal cord. For more caudal (lumbar) regions of the spinal cord, the effect of GABA is less influential on motor neuron development compared to that of glycine ^9^.

 GABA(A) and glycine receptors are the major inhibitory neurotransmitter receptors and contribute to many synaptic functions. Gephyrin promotes recruitment of GABA(A) receptors instead of glycine receptors.  This mechanism could affect the balance of GABA(A) vs. glycine in ALS ^10^.

 Reduced short interval intracortical inhibition that leads to development of cortical hyperexcitability in ALS represents degeneration of inhibitory cortical circuits, combined with excessive excitation of high threshold excitatory pathways. Neuroprotective strategies aimed at preserving the integrity of intracortical inhibitory circuits, in addition to antagonizing excitatory cortical circuits, may provide novel therapeutic targets in ALS ^11^.

 “A wide range of evidence from clinical, histological, genetic, neurophysiological, neuroimaging and neuropsychological studies, suggests that a loss of central nervous system inhibitory neuronal influence is a contributing factor in ALS pathogenesis. *This loss of inhibitory function* points intuitively to an 'interneuronopathy', with natural differences in cortical and spinal inhibitory networks reflected in the hitherto unexplained variable compartmentalization of pathology within upper and lower motor neuron populations. An excitotoxic final common pathway might then result from unopposed glutamatergic activity. If correct, therapies aimed specifically at supporting interneuronal function may provide a novel therapeutic strategy.” ^12^.

 Along with GABA and glycine, the coexpressed interneuron neuromodulatory peptides, like somatostatin, VIP, CCK, and parvalbumin have major influences on the network functions and channel expression. Other neuropeptides also contribute to the complexity. α-MSH preserves GAD67 expression and prevents loss of the somatostatin-expressing subtype of GABAergic GAD67+ inhibitory interneurons ^13^.

**GABAergic Interneurons Contribute to Central Pattern Generators**

 Well controlled pharmacologic manipulation of GABAergic networks may be difficult.

 Central pattern generators are neuronal circuits that when activated can produce rhythmic motor patterns such as walking, breathing, flying, and swimming in the absence of sensory or descending inputs that carry specific timing information ^14^.

 A class of GABAergic sensory neurons contacting the CSF in the vertebrate spinal cord are referred to as CSF-cNs. These cells modulate components of locomotor central pattern generators. CSF-cNs form active GABAergic synapses onto V0-v glutamatergic interneurons, an essential component of locomotor Central Pattern Generators. Spinal GABAergic sensory neurons can tune the excitability of locomotor Central Pattern Generators in a state-dependent manner by projecting onto essential components of the excitatory premotor pool ^15^.

 Motor neurons themselves can control, by retrograde transmission, upstream glutamatergic V2a interneurons that drive locomotion ^16^.

 The lumbar central pattern generator for locomotion selectively modulates the intracellular activity of spinal respiratory neurons engaged in expiration. Medullary respiratory network activity of both thoracolumbar expiratory motor neurons and interneurons is rhythmically modulated with hindlimb ipsilateral flexor locomotor activity. Locomotion and respiration must to be tightly coordinated to reduce muscular interferences and facilitate breathing rate acceleration during exercise ^17^.

**Conclusion**

 The question remains. Can we identify a safer, more predictable, therapeutic regimen than PENG/Hydrocortisone by trying to understand which inhibitory interneuron networks require interventions and which do not? And which specific antagonist (or agonist) drugs would optimize our test of the hypothesis? Can specific peptide neuromodulators contribute to improving the regimen? These considerations do not preclude the importance of carefully testing the PENG/Hydrocortisone regimen itself. The complexity of this task alone is significant. Refinements of the regimen only add complexity, but may make the difference between safe, practical use in ALS and early rejection of the approach.

I have read this submission. I believe that I have an appropriate level of expertise to confirm that it is of an acceptable scientific standard.

## References

[1] O'LearyTWilliamsAHFranciAMarderE: Cell types, network homeostasis, and pathological compensation from a biologically plausible ion channel expression model. *Neuron*.2014;82(4) : 10.1016/j.neuron.2014.04.002 809-21 10.1016/j.neuron.2014.04.002 24853940PMC4109293

[2] HeMTucciaroneJLeeSNigroMJKimYLevineJMKellySMKrugikovIWuPChenYGongLHouYOstenPRudyBHuangZJ: Strategies and Tools for Combinatorial Targeting of GABAergic Neurons in Mouse Cerebral Cortex. *Neuron*.2016;91(6) : 10.1016/j.neuron.2016.08.021 1228-43 10.1016/j.neuron.2016.08.021 27618674PMC5223593

[3] TedeschiADuprazSLaskowskiCJXueJUlasTBeyerMSchultzeJLBradkeF: The Calcium Channel Subunit Alpha2delta2 Suppresses Axon Regeneration in the Adult CNS. *Neuron*.2016;92(2) : 10.1016/j.neuron.2016.09.026 419-434 10.1016/j.neuron.2016.09.026 27720483

[4] PetriSKolleweKGrotheCHoriADenglerRBuflerJKrampflK: GABA(A)-receptor mRNA expression in the prefrontal and temporal cortex of ALS patients. *J Neurol Sci*.2006;250(1-2) : 10.1016/j.jns.2006.08.005 124-32 10.1016/j.jns.2006.08.005 17011586

[5] FoersterBRPomperMGCallaghanBCPetrouMEddenRAMohamedMAWelshRCCarlosRCBarkerPBFeldmanEL: An imbalance between excitatory and inhibitory neurotransmitters in amyotrophic lateral sclerosis revealed by use of 3-T proton magnetic resonance spectroscopy. *JAMA Neurol*.2013;70(8) : 10.1001/jamaneurol.2013.234 1009-16 10.1001/jamaneurol.2013.234 23797905PMC4382938

[6] Nieto-GonzalezJLMoserJLauritzenMSchmitt-JohnTJensenK: Reduced GABAergic inhibition explains cortical hyperexcitability in the wobbler mouse model of ALS. *Cereb Cortex*.2011;21(3) : 10.1093/cercor/bhq134 625-35 10.1093/cercor/bhq134 20643756

[7] ChangQMartinLJ: Glycinergic innervation of motoneurons is deficient in amyotrophic lateral sclerosis mice: a quantitative confocal analysis. *Am J Pathol*.2009;174(2) : 10.2353/ajpath.2009.080557 574-85 10.2353/ajpath.2009.080557 19116365PMC2630565

[8] MartinLJChangQ: Inhibitory synaptic regulation of motoneurons: a new target of disease mechanisms in amyotrophic lateral sclerosis. *Mol Neurobiol*.2012;45(1) : 10.1007/s12035-011-8217-x 30-42 10.1007/s12035-011-8217-x 22072396PMC3530198

[9] FogartyMJSmallcombeKLYanagawaYObataKBellinghamMCNoakesPG: Genetic deficiency of GABA differentially regulates respiratory and non-respiratory motor neuron development. *PLoS One*.2013;8(2) : 10.1371/journal.pone.0056257 e56257 10.1371/journal.pone.0056257 23457538PMC3574162

[10] MaricHMKasaragodVBHausratTJKneusselMTretterVStrømgaardKSchindelinH: Molecular basis of the alternative recruitment of GABA(A) versus glycine receptors through gephyrin. *Nat Commun*.2014;5: 10.1038/ncomms6767 5767 10.1038/ncomms6767 25531214

[11] VucicSCheahBCKiernanMC: Defining the mechanisms that underlie cortical hyperexcitability in amyotrophic lateral sclerosis. *Exp Neurol*.2009;220(1) : 10.1016/j.expneurol.2009.08.017 177-82 10.1016/j.expneurol.2009.08.017 19716820

[12] TurnerMRKiernanMC: Does interneuronal dysfunction contribute to neurodegeneration in amyotrophic lateral sclerosis?. *Amyotroph Lateral Scler*.2012;13(3) : 10.3109/17482968.2011.636050 245-50 10.3109/17482968.2011.636050 22424125

[13] MaKMcLaurinJ: α-Melanocyte stimulating hormone prevents GABAergic neuronal loss and improves cognitive function in Alzheimer's disease. *J Neurosci*.2014;34(20) : 10.1523/JNEUROSCI.5075-13.2014 6736-45 10.1523/JNEUROSCI.5075-13.2014 24828629PMC6608113

[14] MarderEBucherD: Central pattern generators and the control of rhythmic movements. *Current Biology*.2001;11(23) : 10.1016/S0960-9822(01)00581-4 R986-R996 10.1016/S0960-9822(01)00581-4 11728329

[15] FidelinKDjenouneLStokesCPrendergastAGomezJBaradelADel BeneFWyartC: State-Dependent Modulation of Locomotion by GABAergic Spinal Sensory Neurons. *Curr Biol*.2015;25(23) : 10.1016/j.cub.2015.09.070 3035-47 10.1016/j.cub.2015.09.070 26752076

[16] SongJAmpatzisKBjörnforsEREl ManiraA: Motor neurons control locomotor circuit function retrogradely via gap junctions. *Nature*.2016;529(7586) : 10.1038/nature16497 399-402 10.1038/nature16497 26760208

[17] Le GalJPJuvinLCardoitLMorinD: Bimodal Respiratory-Locomotor Neurons in the Neonatal Rat Spinal Cord. *J Neurosci*.2016;36(3) : 10.1523/JNEUROSCI.1825-15.2016 926-37 10.1523/JNEUROSCI.1825-15.2016 26791221PMC6602009

# Peer review: Referee response for version 1

**DOI:** 10.5256/f1000research.10034.r15388

**Published:** 2016-08-22

**Authors:** Pieter Gaillard

**Affiliations:** 1 2-BBB Medicines BV, Leiden, Netherlands

Dr Bert Tuk has provided the readers with a fascinating Opinion Article, in which he sets out his hypothesis that syphilitic ALS is actually not caused by syphilis. And as a result, the pharmacotherapy that was used to treat and cure the confounding syphilitic origin of the ALS symptoms may offer the first effective treatment option for patients suffering from the fatal disease ALS, be it with or without the presence of syphilis.   

 This Opinion Article builds on the earlier presented Opinion Article in this journal by Dr. Tuk, entitled: Inhibitory system overstimulation plays a role in the pathogenesis of neuromuscular and neurological diseases: a novel hypothesis ^1^. In this paper he discloses his assessment of a fundamentally new view of how the symptoms of ALS could be caused, essentially by an overstimulation of the GABA neurotransmitter system, and his current paper basically provided case study proof of concept for this hypothesis with a specific treatment regimen of existing drugs (penicillin G and hydrocortisone) that may indeed act to normalise this overstimulated GABA neurotransmitter system.

 The methods and analysis of the existing literature and data are sound, as is the discussion leading to the new hypothesis, although a more direct link and explanation to the suggested mechanism (modification of the overstimulation of the GABA neurotransmitter system) would have been helpful to the reader.

 And a few more words of caution could have been added to the discussion section, since this work will likely result in a “call to action” by patients and their treating physicians. It is therefore important to advise to keep tight involvement of expert (clinical) pharmacologists, neurologists, and neuroscientists as it is yet unknown what the impact of any modification to this specific treatment regimen would do to an individual patient. Today we don’t know how strict the applied pharmacotherapeutic regimen is to render the expected effect. Can the regimen be modified, or improved? Will modification in the regimen result in any effects on tolerance development, or perhaps sensitization, with risk of seizure activity? How will the two drugs (penicillin G and hydrocortisol) interact when the regimen is modified? Can other compounds be used with a similar pharmacologic profile? Definitely one cannot simply replace to other antibiotics, like minocycline, which was proven effective as alternative in treating neurosyphilis ^2^, yet in ALS patients proven to be detrimental ^3^, perhaps - according the hypothesis of Dr. Tuk -because minocycline has GABA agonistic properties ^4^. Many key questions that deserve careful consideration, monitoring and further studies. Other domains that require further attention is the role of the bulbar onset and treatment effect, since this patient seemed less responsive. And since the two drugs effect two interconnected major systems in the body simultaneously (penicillin G on the inhibitory neurotransmitter system and hydrocortisol on the endocrinology system, both centrally and systemically), there is a world of knowledge to be gained in ALS and also other related diseases how this interaction will play out, leading to hopefully to the long awaited improvements for these devastating brain diseases.

I have read this submission. I believe that I have an appropriate level of expertise to confirm that it is of an acceptable scientific standard.

## References

[1] TukB: Overstimulation of the inhibitory nervous system plays a role in the pathogenesis of neuromuscular and neurological diseases: a novel hypothesis. *F1000Research*.2016;5: 10.12688/f1000research.8774.2 10.12688/f1000research.8774.2 PMC498448127547379

[2] De MariaASolaroCAbbruzzeseMPrimaveraA: Minocycline for symptomatic neurosyphilis in patients allergic to penicillin. *N Engl J Med*.1997;337(18) : 10.1056/NEJM199710303371817 1322-3 10.1056/NEJM199710303371817 9380090

[3] GordonPHMooreDHMillerRGFlorenceJMVerheijdeJLDoorishCHiltonJFSpitalnyGMMacArthurRBMitsumotoHNevilleHEBoylanKMozaffarTBelshJMRavitsJBedlackRSGravesMCMcCluskeyLFBarohnRJTandanR: Efficacy of minocycline in patients with amyotrophic lateral sclerosis: a phase III randomised trial. *Lancet Neurol*.2007;6(12) : 10.1016/S1474-4422(07)70270-3 1045-53 10.1016/S1474-4422(07)70270-3 17980667

[4] AhmadiradNShojaeiAJavanMPourgholamiMHMirnajafi-ZadehJ: Effect of minocycline on pentylenetetrazol-induced chemical kindled seizures in mice. *Neurol Sci*.2014;35(4) : 10.1007/s10072-013-1552-0 571-6 10.1007/s10072-013-1552-0 24122023
